# Spatiotemporal and demographic variation in the diet of New Zealand lesser short‐tailed bats (*Mystacina tuberculata*)

**DOI:** 10.1002/ece3.4268

**Published:** 2018-07-09

**Authors:** Zenon J. Czenze, J. Leon Tucker, Elizabeth L. Clare, Joanne E. Littlefair, David Hemprich‐Bennett, Hernani F. M. Oliveira, R. Mark Brigham, Anthony J. R. Hickey, Stuart Parsons

**Affiliations:** ^1^ School of Biological Sciences University of Auckland Auckland New Zealand; ^2^ School of Biological and Chemical Sciences Queen Mary University of London London UK; ^3^ Department of Biology University of Regina Regina SK Canada; ^4^ Present address: School of Earth, Environmental and Biological Sciences Queensland University of Technology Brisbane QLD Australia

**Keywords:** Chiroptera, insectivores, molecular diet analysis, *Mystacina tuberculata*, spatial/temporal variation

## Abstract

Variation in the diet of generalist insectivores can be affected by site‐specific traits including weather, habitat, and season, as well as demographic traits such as reproductive status and age. We used molecular methods to compare diets of three distinct New Zealand populations of lesser short‐tailed bats, *Mystacina tuberculata*. Summer diets were compared between a southern cold‐temperate (Eglinton) and a northern population (Puroera). Winter diets were compared between Pureora and a subtropical offshore island population (Hauturu). This also permitted seasonal diet comparisons within the Pureora population. Lepidoptera and Diptera accounted for >80% of MOTUs identified from fecal matter at each site/season. The proportion of orders represented within prey and the Simpson diversity index, differed between sites and seasons within the Pureora population. For the Pureora population, the value of the Simpson diversity index was higher in summer than winter and was higher in Pureora compared to Eglinton. Summer Eglinton samples revealed that juvenile diets appeared to be more diverse than other demographic groups. Lactating females had the lowest dietary diversity during summer in Pureora. In Hauturu, we found a significant negative relationship between mean ambient temperature and prey richness. Our data suggest that *M. tuberculata* incorporate a narrower diversity of terrestrial insects than previously reported. This provides novel insights into foraging behavior and ecological interactions within different habitats. Our study is the first from the Southern Hemisphere to use molecular techniques to examine spatiotemporal variation in the diet of a generalist insectivore that inhabits a contiguous range with several habitat types and climates.

## 1 INTRODUCTION

To maintain homeostasis, individuals must balance energetic transactions (i.e., energy spent vs. energy gained). Expended energy is partitioned between movement, physiological maintenance, somatic growth, and reproduction. Although foraging is an expenditure, it is also the source of energy gain. An individual's energy balance is influenced by biotic and abiotic factors such as photoperiod, food/water availability, digestibility and abundance of prey, and ambient temperature (*T*_*a*_) (Doucette, Brigham, Pavey, & Geiser, 2012; Körtner & Geiser, 2000; McNab, 2002; Song & Geiser, 1997).

Endotherms (i.e., most mammals and birds) face a heavy energetic burden, as the majority of their output is lost as metabolic heat maintaining normothermic body temperatures. Due to surface to volume ratio laws, active small insectivorous endotherms are likely under even greater pressures during winter than similarly sized herbivorous species, as insect populations are more sensitive to weather. For example, many volant insects have limited flight capacities, cannot fly, or are dormant at low *T*_*a*_ (Jones, Duvergé, & Ransome, 1995).

Small endothermic species with ranges distributed across climatic zones likely experience location‐dependent influences to their energetic balance (Dunbar & Brigham, 2010; Stawski & Geiser, 2011; Zervanos, Maher, Waldvogel, & Florant, 2010). Both energy expenditure (i.e., thermoregulatory costs and foraging costs) and energy intake (i.e., prey availability and dietary selection) likely differ seasonally and between habitats. For aerial insectivores, foraging costs are negatively correlated with *T*_*a*_ (Humphries & Careau, 2011; Klüg‐Baerwald, Gower, Lausen, & Brigham, 2016). Insect diversity also correlates with many factors including plant diversity and latitude (Rohde, 1992; Zhang et al., 2016). Therefore, individuals of the same species that inhabit different habitats are likely to differ in energetic expenditure and/or intake (Dunbar & Brigham, 2010).

Many bat species exhibit dietary plasticity and their diet may vary with individual energetic requirements, food availability, season, region, and life‐history stage (Adams, 1996, 1997; Aldridge & Rautenbach, 1987; Anthony & Kunz, 1977; Hermanson & O'Shea, 1983; Johnston & Fenton, 2001; Levin, Yom‐Tov, & Barnea, 2009; O'Shea & Vaughan, 1977). Aspects of an adult bat's life history, such as reproduction and lactation come with higher energetic burdens, and many bat species synchronize lactation with peaks in summer insect diversity (Clare, Symondson, & Fenton, 2014; Clare, Symondson, Broders, et al., 2014; Levin et al., 2009). The greater mouse‐tailed bat (*Rhinopoma microphyllum*) lactation period coincides with the brief periodic nuptial flights of fat‐rich ants (*Camponotus* spp.) on which the bats feed exclusively (Levin et al., 2009). Further, many juvenile insectivorous bats have more varied diets while they learn to fly and hunt, compared to adults (Adams, 1996, 1997; Hamilton & Barclay, 1998; Rolseth, Koehler, & Barclay, 1994).

The New Zealand lesser short‐tailed bat (*Mystacina tuberculata*) is a small forest‐dwelling species, the only extant member of the family Mystacinidae, and is endemic to New Zealand. The species ranges from Omahuta‐Puketi Forest in the North Island (35°13′38.5″S, 173°38′18.31″E) to Whenua Hou/Codfish Island in the South (46°46′23.9″, S167°37′55.7″E) (Carter & Riskin, 2006). Although *M. tuberculata* is omnivorous, they use a combination of aerial hawking and terrestrial foraging to capture arthropods, which make up the majority of their diet (Arkins, Winnington, Anderson, & Clout, 1999; Jones, Webb, Sedgeley, & O'Donnell, 2003; O'Donnell, Christie, Corben, Sedgeley, & Simpson, 1999; Parsons, 1997; Webb, Sedgeley, & O'Donnell, 1998). Microscopic prey identification indicates that *M. tuberculata* predominantly feed on five orders of arthropods: Coleoptera, Lepidoptera, Diptera, Blattodea, and Orthoptera (Arkins et al., 1999). Furthermore, diet appears to change seasonally, with higher proportions of volant insects consumed during summer.

On Little Barrier Island/Hauturu (Hauturu), where the climate is less seasonal, *M. tuberculata* reportedly has the highest dietary diversity during summer (Arkins et al., 1999). Although seasonal variation in diet is apparent and *M. tuberculata* is purported to be an opportunistic forager, these conclusions were based on data for one population and using traditional morphological techniques that may have a bias toward the detection of harder‐bodied, larger prey. Molecular techniques are increasingly used to identify prey and are effective when applied to a generalist foraging species (Clare, Fraser, Braid, Fenton, & Hebert, 2009) and may be particularly effective at identifying small soft‐bodied prey making it an excellent complementary technique.

*Mystacina tuberculata* presents an opportunity to examine spatiotemporal variation in diet because it is a small generalist insectivore with a habitat range that includes different forest types, with access to presumably different insect communities. Using fecal samples collected from bats, we analyzed diet to test several research questions. First, we assessed the variability of *M. tuberculata* diet across New Zealand, to examine whether spatiotemporal variation in resource use is an important form of nutritional or dietary flexibility that is adaptive when resource availability fluctuates. Specifically, we predicted that (a) during the same season, populations from lower latitudes will eat a more diverse diet, (b) bats will have a more diverse diet during summer compared to winter, (c) within a season, prey abundance and diversity will be correlated with *T*_*a*_. Our second research question was to determine whether prey consumption will differ due to demographic differences in energy demands, such as that between lactating females and nonreproductive adults. Furthermore, we predicted that juveniles would consume a more diverse diet assuming they have naïve foraging behavior.

## 2 METHODS

All procedures were approved by the University of Auckland Animal Ethics Committee (AEC‐R1374) and were conducted under New Zealand Department of Conservation Wildlife Act Authorization Number 39083‐FAU. Our study was conducted at three sites: (a) the Pikiariki Ecological Area of Pureora Forest Park (Pureora; 38°26′S, 175°39′E), central North Island, New Zealand, during January 2014–April 2015 (Pureora summer) and May–July 2015 (Pureora winter); (b) the Eglinton Valley of Fiordland National Park (Eglinton; 44°58′S, 168°00′E), South Island, New Zealand, during January–April 2016 (Eglinton); and (c) Hauturu/Little Barrier Island (Hauturu), 80 km off the east coast of the North Island, New Zealand, during May–July 2016 (Hauturu).

Pureora Forest Park is a mature podocarp–hardwood forest containing kahikatea (*Dacrycarpus dacrydioides*), mataī (*Prumnopitys taxifolia*), miro (*Prumnopitys ferruginea*), rimu (*Dacrydium cupressinum*), and tōtara trees (*Podocarpus tōtara*). The forest is characterized by a low canopy with a dense understory and bordered by exotic pine plantations and pastoral land. The Eglinton Valley is dominated by a temperate southern beech forest consisting of red (*Nothofagus fusca*) and silver beech (*N. menziesii*). Hauturu is the only large forested area in New Zealand relatively unaffected by introduced browsing mammals. pōhutukawa (*Metrosideros excelsa*), kohekohe (*Dysoxylum spectabile*), puriri (*Vitex lucens*), taraire (*Beilschmiedia tarairi*), kauri (*Agathis australis*), northern rātā (*Metrosideros robusta*), tawheowheo (*Quintinia serrata*), tawari (*Ixerba brexioides*), and southern rātā (*Metrosideros umbellata*) trees are common.

Bats were captured using harp traps and mist nets. Individuals were weighed to the nearest 0.5 g using a Pesola spring scale (Pesola AG, Schindellegi, Switzerland). We recorded sex and measured forearm length to the nearest 1 mm. Adult females were classified as nonreproductive (no obvious bare patches around the nipples), pregnant (determined through gentle abdominal palpation), lactating (large bare nipples and milk produced when pressed), and postlactating (nipples visible but no milk could be expressed). Juvenile bats were distinguished from adults by the lack of ossification of the metacarpal–phalangeal joint on the third digit (Racey, 1974). After measurement, individuals were held singly in cloth bags for up to 1 hr or until they defecated. Fecal samples (all pellets collected per bat) were stored at −20°C in 1.7‐ml microcentrifuge tubes.

The QIAamp Stool Mini Kit (Qiagen, UK) was used to extract DNA from fecal samples from individual bats following the manufacturer's instructions, but including modifications suggested by Zeale, Butlin, Barker, Lees, and Jones (2011) and Clare, Symondson, and Fenton (2014). PCR and sequencing were performed by the Genome Centre (Queen Mary University of London). In brief: Amplification of a 157‐bp fragment of the mitochondrial cytochrome c oxidase subunit 1 was performed using primers ZBJ‐ArtF1c and ZBJ‐ArtR2c (Zeale et al., 2011) adapted to include Fluidigm tags CS1 and CS2. Each 10 μl PCR contained 5 μl of Qiagen multiplex PCR (Qiagen, CA) master mix, 3 μl of water, 0.5 μl of each 10 μM primer, and 1 μl of eluted DNA. PCR amplification was as follows: 95°C, 15 min; 50 cycles of 95°C, 30 s; 52°C, 30 s; 72°C, 30 s, and 72°C, 10 min. These primers may be biased toward Lepidoptera, but still accurately reflect the preference for beetles as the dominant group recovered in an analysis of beetle specialists (Clare, Symondson, & Fenton, 2014). Amplicon QC was performed using a DNA D1000 TapeStation (Agilent Technologies), and quantification was performed using a QuBit dsDNA HS Assay Kit (Invitrogen, Life Technologies). Sequencing was performed bidirectionally with 10‐bp Fluidigm indexes following manufacturer's protocols, and sequencing was run on the MiSeqv2 Chemistry using a 2 × 150 bp run with 300 cycle run (Illumina).

Reads were merged in Mothur (Schloss et al., 2009) and then processed using the Galaxy platform (Blankenberg, Von Juster, & Coraor, 2010; Giardine et al., 2005; Goecks, Nekrutenko, Taylor, & Galaxy Team, 2010). Primer sequences were removed and all sequences that were longer or shorter than the target amplicon length of 157 bp were filtered out. Sequences were collapsed into unique haplotypes, and then, singleton sequences were excluded from further analyses. Sequences were clustered into molecular operational taxonomic units (MOTU; Floyd, Abebe, Papert, & Blaxter, 2002), and a representative sequence of each MOTU was picked for analysis with the QIIME pick otu and uclust methods (http://qiime.sourceforge.net; Caporaso et al., 2010). MOTU were clustered using a similarity threshold of 94% to minimize spurious OTU generation (see Clare, Chain, Littlefair, Cristescu, & Deiner, 2016 for the appropriateness of MOTU cluster levels for diet analysis). We identified MOTU to order level using BLAST analyses and a reference database of >600,000 DNA barcodes extracted from GenBank with a wider taxonomic profile (including potential contaminants bacteria, fungi, mammals). MEGAN version 5.6.3. (Huson, Mitra, Ruscheweyh, Weber, & Schuster, 2011) was used to screen out unknowns, unidentified sequences and those not resolved to order with the LCA parameters recommended by Salinas‐Ramos, Herrera Montalvo, León‐Regagnon, Arrizabalaga‐Escudero, and Clare (2015). The remaining identified MOTU were used for statistical analysis of diet.

At each study site, we recorded *T*_*a*_ using data loggers (HOBO Micro Station Data Logger—H21‐002, Onset Computer Corporation, Bourne, MA, USA) placed 2 m above the ground in the shade.

For ecological analysis, we split the data into “winter” (May 1–August 1) and “summer” (January 1–April 1) and examined differences between sites (Hauturu vs. Pureora winter; Eglinton vs. Pureora summer) and seasons (Pureora winter vs. Pureora summer). In total, we collected faces from 243 captured bats (Eglinton: 42, Hauturu: 19, Pureora winter: 29, Pureora summer: 153). To avoid potential confounding variables, ecological analyses were restricted to adult males and nonreproductive adult females (Eglinton: 22, Hauturu: 18, Pureora winter: 14, Pureora summer: 33). For ecological analysis, we removed MOTU for orders that bats do not intentionally eat (e.g., nematodes, most likely parasites from the bat's or prey's gut). We conducted ecological analyses in PAST (Hammer, Harper, & Ryan, 2001) on order‐level data and compared the value of Simpson's diversity indices among locations and seasons with *p*‐values estimated by bootstrapping with 2,000 replicates. We compared the proportion of occurrence of each order in the diet (proportion = number of MOTU in an order/total number of MOTU, where MOTU is a proxy for species) among locations and sampling periods using a *χ* ^2^ frequency test with *p‐*values computed using a Monte Carlo simulation with 2,000 replicates using R (v. 3.4.0; R Development Core Team, 2014).

We compared MOTU richness (number of MOTU present in a fecal sample) between sites using a Kruskal–Wallis H test, or a one‐way ANOVA (if data were hetero‐ or homoscedastic, respectively), followed by a *post hoc* Tukey HSD test to generate specific *p*‐values using R (v. 3.4.0; R Development Core Team, 2014). As we found no difference in MOTU richness at any site between juvenile males and juvenile female, data were pooled for further analysis. We also found no differences between adult males and nonreproductive females at any site, so their data were also pooled. We performed linear regression using linear models (packages “nlme,” “lme4,” and “MuMIn” in R v. 3.4.0, R Development Core Team, 2014) to analyze data with MOTU richness as a dependent variable and demographic (e.g., juvenile, nonreproductive adult, lactating adult female), date, and mean night *T*_*a*_ as independent variables. We conducted model selection by comparing models, starting with a saturated model including the interaction of all explanatory variables, using maximum likelihood tests until only significant variables remained.

We followed Razgour et al. (2011), and the extent of dietary specialization was determined at the MOTU level using the standardized Levins’ measure of niche breadth $B=\frac{1}{\sum {P_{i}}^{2}}$ standardized as $B_{A}=\frac{B-1}{n-1}$ where *B* is Levins’ measure, *P*_*i*_ is the proportion of fecal samples in which MOTU *i* was found, and *n* is the number of possible MOTUs in the diet.

We quantified dietary resource overlap at the MOTU level among seasons, demographic, and sites using Pianka's (Pianka, 1973) measure of niche overlap $O_{\mathrm{jk}}=\frac{\sum_{i}^{n}P_{\mathrm{ij}}P_{\mathrm{ik}}}{\sqrt{\sum_{i}^{n}P_{\mathrm{ij}}^{2}\sum_{i}^{n}P_{\mathrm{ik}}^{2}}}$ where *P*_*ij*_ is the proportion that resource *i* is of the total resources used by group *j*;* P*_*ik*_ is the proportion that resource *i* is of the total resources used by group *k*; and *n* is the total number of resource states (total number of MOTUs). Null models were used to test whether the extent of niche overlap is greater than expected by chance, and determine the effect of season and sex on dietary resource use. We generated 1,000 simulated matrices of randomized MOTU diet composition, using the software EcoSim (version 7; http://grayentsminger.com/ecosim.htm) with randomization algorithm 3, and compared observed and randomly simulated extents of niche overlap. We assed significance at *p < *0.05.

## 3 RESULTS

Analyses were conducted on fecal matter collected between November 14, 2014, and June 28, 2016, from 243 individual bats (adult male: 106; lactating females: 55; nonreproductive adult females: 56; juvenile females: 15; juvenile males: 11). We found 1,006 unique MOTU from 17 probable prey orders (Table 1). The diet of bats at all sites and in both seasons was dominated by MOTU identified as Lepidoptera (63%–81%) or Diptera (8%–18%).

**Table 1:** Order‐level taxonomic diversity of prey items in *Mystacina tuberculata* feces (*N* = 243) collected between 2014 and 2017 from Pureora, Eglinton, and Hauturu, New Zealand

Order	No. MOTU	% Frequency of occurrence
Araneae	40	2.57
Blattodea	5	2.14
Coleoptera	45	4.30
Collembola	8	0.23
Decapoda	8	0.31
Diptera	197	12.79
Ephemeroptera	5	0.67
Hemiptera	16	0.69
Hymenoptera	6	0.15
Lepidoptera	656	74.88
Mantodea	1	0.03
Neuroptera	4	0.39
Orthoptera	5	0.15
Plecoptera	2	0.28
Psocoptera	3	0.28
Scolopendromorpha	1	0.03
Trichoptera	3	0.10

*Note*

No. MOTU is the number of distinct MOTUs found in all fecal samples. % Frequency of occurrence is the number of occurrences from the order/total number of occurrences for all fecal samples multiplied by 100.

**Figure 1:**
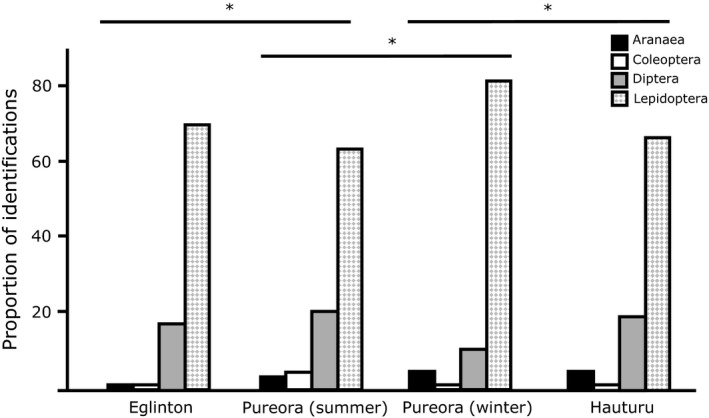
Diversity in prey consumed by adult nonreproductive *Mystacina tuberculata*. The proportion of each prey group in the diet varied significantly between sites and seasons. (*N* = 22 [Eglinton]; 33 [Pureora summer]; 14 [Pureora winter]; 18 [Hauturu]). Proportion = number of MOTU of that order/total number of MOTU. * indicates *p *<* *0.05

**Figure 2:**
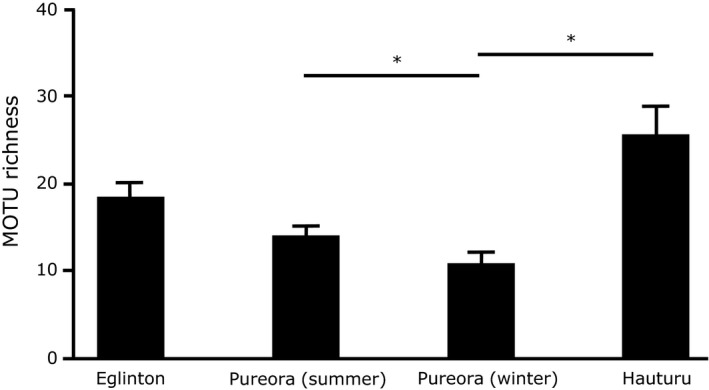
Mean prey MOTU richness within adult nonreproductive *Mystacina tuberculata* fecal samples, based on data restricted to ordinal‐level taxonomy. (*N* = 22 [Eglinton]; 33 [Pureora summer]; 14 [Pureora winter]; 18 [Hauturu]). * represents a significant difference *p *<* *0.05; bars represent ± *SE* mean

Using order‐level taxonomy of prey from nonreproductive adults only, the proportion of orders consumed and the value of the Simpson diversity indices of diet differed between winter sites, summer sites, and seasonally in Pureora (Figure 1, Table 2). Dietary MOTU richness (number of distinct MOTU in a sample) varied between sites (*df *= 3, 83; *F *=* *5.9; *p *<* *0.01; Figure 2). Post hoc analysis revealed differences between winter sites with Hauturu bats having greater MOTU richness than bats in Pureora. Pureora bats had higher MOTU richness in summer than in winter but not compared to individuals from Eglinton during summer.

**Table 2:** Comparisons of chi‐square, Simpson diversity index, MOTU richness, niche breadth (Levin's adjusted *B*), and Pianka's measure of niche overlap (*O*_*jk*_) between populations of nonreproductive adult *Mystacina tuberculata* from winter sites (Hauturu and Pureora), summer sites (Eglinton and Pureora), and seasonally in Pureora New Zealand

	χ^2^	*p*‐Value	Simpson diversity index	*p*‐Value	MOTU richness	*p*‐Value	Niche breadth	Niche overlap	*p*‐Value
Winter	9.5	0.03	Hauturu = 0.52 (*N* = 18) Pureora = 0.32 (*N* = 14)	<0.01	Hauturu 25.8 ± 3.2 Pureora 8.2 ± 2.0	<0.01	Hauturu 0.09 Pureora 0.047	0.26	0.5
Summer	9.35	0.03	Eglinton = 0.46 (*N* = 22) Pureora = 0.55 (*N* = 33)	0.02	Eglinton 16.6 ± 2.2 Pureora 18.6 ± 2.3	0.9	Eglinton 0.092 Pureora 0.15	0.62	<0.01
Pureora	17.3	<0.01	Winter = 0.32 (*N* = 14) Summer = 0.55 (N = 33)	<0.01	Winter 8.2 ± 2.0 Summer 18.6 ± 2.3	0.035	Winter 0.047 Summer 0.15	0.35	<0.01

*Note*

MOTU richness is the ±*SE* mean. Each comparison is followed by the respective *p*‐Value.

During summer in Pureora, there were differences among demographic classes (*N* = 40 (lactating females); 33 (nonreproductive adults); 11 (juveniles); *df *= 2, 81, *F *=* *5.0, *p *<* *0.01) with lower MOTU richness in lactating females compared to nonreproductive adults (Table 3).

**Table 3:** Comparisons of MOTU richness among demographics of *Mystacina tuberculata* from Pureora and Eglinton, New Zealand. *p*‐Values were generated from a Tukey's HSD test

		Lactating female	NR Adult	Juvenile	*p*‐value
Pureora (summer)	MOTU richness	9.8 ± 1.1 (*N* = 40)	17.1 ± 2.1 (*N* = 33)	17.6 ± 3.4 (*N* = 11)	L versus NR = 0.09 L versus J = 0.01 J versus NR = 0.97
Eglinton	MOTU richness	16.1 ± 6.7 (*N* = 9)	16.6 ± 2.2 (*N* = 22)	34.7 ± 5.4 (*N* = 7)	L versus NR = 0.16 L versus J = 0.03 J versus NR = 0.01
Pureora (winter)	MOTU richness	NA	8.8 ± 2.1 (*N* = 14)	16.2 ± 4.0 (*N* = 6)	J versus NR = 0.09

*Note*

J: juvenile; L: lactating; NR: nonreproductive.

During summer in Eglinton, the MOTU richness differed among demographic classes (*N* = 9 (lactating females); 22 (nonreproductive adults); 7 (juveniles); *df *= 2, 35, *F *=* *4.98, *p *=* *0.01) with higher MOTU richness in juveniles relative to lactating females and nonreproductive adults (Table 3).

During winter in Pureora, there were no differences between juveniles and nonreproductive adults in MOTU richness (*N* = 14 (nonreproductive adults); 6 (juveniles); *df *= 1, 18, *F *=* *3.27, *p *=* *0.09).

**Figure 3:**
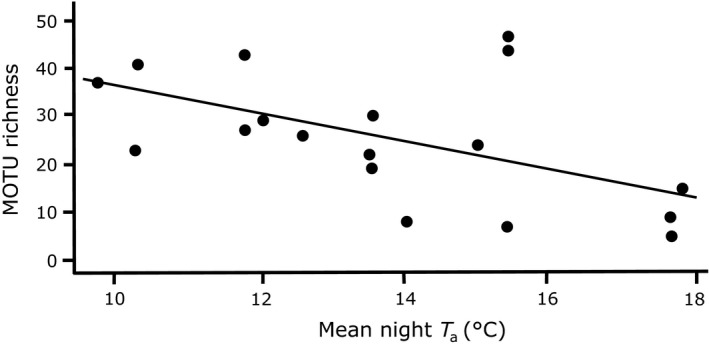
Prey MOTU richness from nonreproductive adult *Mystacina tuberculata* fecal samples as a function of mean nightly *T*_*a*_ for individuals from Hauturu, New Zealand. MOTU richness decreased with increasing *T*_*a*_ (*df* = 16, *T *=* *−2.2*, R* ^2^ = 0.24, *p *=* *0.04)

We only caught nonreproductive adults in Hauturu so we were unable to compare demographics. However, this was the only site where there was a relationship between mean nightly *T*_*a*_ and MOTU richness (*N* = 18; *df *= 17, *T *=* *−2.2*, R* ^2^ = 0.24, *p *=* *0.04; Figure 3).

Analysis carried out at the MOTU level showed that nonreproductive adults at all sites had relatively narrow niches (Table 2). Individuals from Pureora during summer (*B*_*A*_ **= 0.15) had the broadest niche and individuals from Pureora during winter (*B*_*A*_ **= 0.05) the narrowest. Despite being captured during winter, individuals from Hauturu had a similar niche breadth (*B*_*A*_ **= 0.09) to individuals from summer in Eglinton (*B*_*A*_ **= 0.09).

During summer in Pureora, nonreproductive adults had the broadest niche breadth (*B*_*A*_ = 0.15), followed by lactating females (*B*_*A*_ = 0.076), and juveniles (*B*_*A*_ = 0.067). However, in winter, juveniles niche was broadest (*B*_*A*_ = 0.07) followed by nonreproductive adults (*B*_*A*_ = 0.047).

During summer in Eglinton, juveniles had the broadest niche (0.11), followed by nonreproductive adults (*B*_*A*_ = 0.09), and lactating females (*B*_*A*_ = 0.07).

For nonreproductive adults, dietary niche, measured based on MOTUs, significantly overlapped for all sites (Table 2) except for individuals from Hauturu and Pureora during winter (*O*_*jk*_ = 0.26, *p* = 0.5).

We found that dietary niche overlapped among demographic classes at all sites (Table 4) except for juveniles and nonreproductive adults in Pureora during winter (*O*_*jk*_ = 0.55, *p* = 0.72) and juveniles and lactating females in Pureora during summer (*O*_*jk*_ = 0.3, *p* = 0.23).

**Table 4:** Comparisons of Pianka's niche overlap among nonreproductive (NR) adults, juveniles, and lactating females of *Mystacina tuberculata* from Pureora and Eglinton, New Zealand. Comparisons took place in summer in Purora (PS), and Eglinton (ES), and also during winter in Pureora (PW)

	(PS) NR adults versus Juveniles	(PS) NR adults versus Lactating females	(PS) Juveniles versus Lactating females	(PW) NR adults versus Juveniles	(ES) NR adults versus Lactating females	(ES) NR adults versus Juveniles	(PS) Juveniles versus Lactating females
Niche overlap	0.53	0.56	0.3	0.55	0.65	0.72	0.71
*p* Value	0.01	0.01	0.23	0.72	0.01	0.01	0.01

## 4 DISCUSSION

Our study is one of the first to use molecular techniques to examine spatiotemporal variation in the diet of a generalist insectivore that inhabits a contiguous range with several habitat types and climates. We found support for our first hypothesis that diet is affected by site and season. We found that prey orders consumed differed between winter and summer sites as well as seasonally and that diversity was higher in summer compared to winter. However, it was not a summer site that had the highest MOTU richness, but Hauturu during winter. Hauturu was also the only site where there was a significant relationship between mean *T*_*a*_ and diet. There was also support for our second hypothesis that diet differed due to demography. Juveniles had a more diverse diet than any other demographic group and lactating females had the lowest dietary diversity. Our data suggest that, although the fecal samples of *M. tuberculata* are dominated by Lepidoptera and Diptera, several site‐specific seasonal and demographic variables influence diet.

The use of molecular dietary analysis and the consideration involved in the analyses of these data have been discussed previously (e.g., Clare et al., 2016; Pompanon et al., 2012). There are two important issues in interpreting the data we collected. First, we detected an unexpectedly high proportion of Lepidoptera and Diptera. Traditional morphologically based analyses have suggested these bats eat more Coleoptera (beetles) and terrestrial insects (Arkins et al., 1999). Molecular analyses are more sensitive to small, soft, easily digested material than traditional analyses (Clare et al., 2009) but cannot quantify biomass reliably. The primers employed may also preferentially amplify Lepidoptera (Alberdi, Aizpurua, Gilbert, & Bohmann, 2018). While our analysis may underestimate taxa such as Coleoptera and Orthoptera, the effect is likely not large, as these taxa dominated the diet of a beetle specialist in a study employed the same analysis (e.g., Clare, Symondson, & Fenton, 2014). Second, we used MOTU and a clustering threshold of 94%. This is relatively low compared to the suspected reality of species identified (see Discussion in Clare et al., 2016), but is recommended to reduce MOTU inflation (Clare et al., 2016; Flynn, Brown, Chain, MacIsaac, & Cristescu, 2015). We have used the empirical recommendations of Clare et al. (2016) to be conservative, and note that MOTU should not be equated to “species” (see Floyd et al., 2002) but as a comparable taxonomic entity for ecological and statistical interpretations. All things considered, our results likely give a reliable insight into the insectivorous habits of *M. tuberculata*, although we must note that bats were sampled during the summer and winter months and our results represent only a snapshot of the bats’ dietary habits.

Our study adds items to the list of prey known to be consumed by *M. tuberculata*. In addition to the orders that have been previously reported for *M. tuberculata* (Arkins et al., 1999), we found MOTU of Collembola, Decapoda, Ephemeroptera, Plecoptera, Psocoptera, Scolopendromorpha, and Trichoptera. Further, we found MOTU from several ecto‐ and endoparasite orders Oribatida, Siphonaptera, Astigmata, Rhabditida, Adinetida, Tylenchida.

*Mystacina tuberculata* has a diverse diet and Daniel (1979) reported that *M. tuberculata* feed mainly on Coleoptera, Lepidoptera, Diptera, and Orthoptera. During April–May in Hauturu, Arkins et al. (1999) reported the percentage occurrence (number of samples that contained at least one fragment from an order divided by total number of samples) for Lepidoptera (3%), Coleoptera (46%), Diptera (17%), Orthoptera (66%), and Araneae (31%). When we compare our percentage occurrence (number of samples that contained at least one MOTU from an order divided by total number of samples) for our Hauturu site there is a stark contrast. We detected Lepidopteran DNA in 100% (18/18 individuals) of Hauturu samples. Further, the other main orders also differed; Coleoptera (23%), Diptera (94%), Orthoptera (0%), Araneae (56%). All previous work analyzing *M. tuberculata* diet used visual inspection and morphological analysis of partially digested prey remains in feces (Arkins et al., 1999; Daniel, 1979). This technique has limitations as more hard‐bodied prey will be recognizable after digestion, leading to an over‐representation of these taxa compared to soft‐bodied prey (Nielsen, Clare, Hayden, Brett, & Kratina, 2017). Conversely, molecular analysis has been demonstrated to accurately identify hard‐bodied prey and small, soft‐bodied prey (Clare et al. 2009), but may be biased by primer binding and available reference collections (Nielsen et al., 2017). As such, both methods should be seen as confirming the presence of dietary items with different and potentially complimentary approaches. Further work which combines multiple techniques is suggested.

We report differences in the value of the Simpson diversity index, the proportion of orders consumed, and MOTU richness between summer sites. Individuals from Pureora had a more diverse diet than individuals from Eglinton, and exhibited a higher dietary niche breadth, which suggests a more generalist diet. These sites are separated by 6° of latitude, but Czenze, Brigham, Hickey, and Parsons (2017b) reported that mean summer *T*_*a*_ of each site was within 1°C. Therefore, it is unlikely that the differences we observed were caused by temperature differences. One explanation for the site‐specific difference is the forest type. The Eglinton Valley is dominated by two tree species and has low invertebrate abundance, typical of forests in temperate climates, and the bats inhabiting it have larger home ranges compared to Pureora (O'Donnell et al., 1999). Conversely, Pureora is comprised of several tree species, and individuals from Pureora have a smaller home range compared to Eglinton, suggesting a higher prey abundance and/or diversity (Toth, Cummings, Dennis, & Parsons, 2015). In other parts of the world, there is a positive relationship between and plant and insect diversity (Zhang et al., 2016).

Despite sampling during winter, individuals from Hauturu had the second highest value of the Simpson diversity index, which differed from Pureora individuals. Furthermore, the proportion of orders consumed differed with Hauturu having the highest mean MOTU richness of all sites. Hauturu and Pureora were also the only sites where adults did not have dietary niche overlap. The difference in winter climate between the two sites could partially explain differences. Aerial insect abundance can decrease dramatically with decreasing *T*_*a*_ (Jones et al., 1995), and *T*_*a*_ < 10°C has often been reported to constrain insect abundance (Hope & Jones, 2012; Park, Jones, & Ransome, 2000). During winter, mean night *T*_*a*_ in Hauturu (12.1 ± 2.4°C) is higher than Pureora (6.2 ± 2.7°C), mean night *T*_*a*_ > 10°C occurred on 92% of observation nights in Hauturu compared to 7% in Pureora, and *T*_*a*_ never dropped below 0°C in Hauturu but did so on 26% of nights in Pureora (Czenze, Brigham, Hickey, & Parsons, 2017a). Therefore, the winter conditions in Hauturu are likely to increase the abundance and diversity of flying insects. If *M. tuberculata* are feeding opportunistically, it may also explain the dietary differences. Hauturu is also unique as it is free of mammalian and insect pests. The high diversity and MOTU richness may be due to the pristine nature of the island reserve as non‐native flora and fauna can adversely affect insect diversity (Bezemer, Harvey, & Cronin, 2014; Burghardt & Tallamy, 2015; New, 2016). It may be that, during winter, Hauturu is more suitable for promoting both insect diversity and bat activity than Pureora. Alternatively, if resources are limited, individuals may respond by increasing the abundance of a particular resource or increasing their flexibility and consuming a wider variety of resources (Clare, Symondson, & Fenton, 2014). Pureora is essentially three distinct habitat types, including non‐native pines and pastoral land, and bats potentially forage in each. It would be interesting to determine how much of *M. tuberculata* diet in Pureora is comprised of non‐native species. Future work should also aim to quantify the insect communities from each habitat type to determine their spatiotemporal variation and help elucidate their role in the variation in *M. tuberculata* diet.

In Pureora, we found seasonal differences in the value of the Simpson diversity index, the proportion of orders consumed, and MOTU richness. Further, during winter individuals had the lowest dietary niche breadth of any group suggesting a more specialist diet. Many insect species are dormant, or inactive during winter, and arthropod consumption by bats, like the Indian pygmy bat (*Pipistrellus tenuis*), varies with season (Kunz, de Torrez, Bauer, Lobova, & Fleming, 2011; Whitaker, Issac, Marimuthu, & Kunz, 1999). Daniel (1979) suggested that, during winter, fewer moths are consumed by *M. tuberculata* due to cold temperature. We found that the proportion of Lepidopteran MOTU increased from summer (65%) to winter (76%), while Dipteran MOTU decreased (17%–5%). The decrease in Dipterans was mirrored by an increase in spiders (4%–12%), suggesting that bats may be switching their foraging techniques or that Dipterans are less available during winter. In captivity, *M. tuberculata* partition foraging to 40% terrestrial, 30% aerial hawking, and 30% gleaning (McCartney, Stringer, & Potter, 2007). These findings, particularly the 40% terrestrial foraging, may result from housing bats in a small enclosure and are not consistent with our results, that is, from a natural population. Although we cannot identify the method by which bats captured prey items, we would expect to see a greater proportion of ground dwelling insects in the diet if these proportions were correct. Future studies could employ accelerometers on free‐ranging bats to determine the partition of foraging between terrestrial, aerial hawking, and gleaning.

On Hauturu, we found a negative relationship between *T*_*a*_ and mean MOTU richness with higher MOTU richness during colder nights compared to warmer nights. The thermoregulatory behavior of bats on Hauturu is influenced more by temperature than mainland bats (Czenze, Brigham, Hickey, & Parsons, 2017c). Although heat produced through activity is used for thermoregulation in a wide range of animals, generally, the costs of flight increases with decreasing *T*_*a*_ (Humphries & Careau, 2011; Klüg‐Baerwald et al., 2016). Further, there is a threshold *T*_*a*_ where flying insects likely become absent, and Czenze et al. (2017c) argued that bats are using the warm *T*_*a*_ as a proxy for the increased probability of foraging success. Insect diversity falls after summer, and big brown bats (*Eptesicus fuscus*) may compensate by increasing their dietary diversity (Clare, Symondson, & Fenton, 2014). If bats choose to forage during colder nights they will expend more energy and, to mitigate these increased costs, cannot afford to be selective. During a warmer evening, individuals are likely to be less energetically burdened and may invest more time foraging to capture higher‐quality prey items.

Alternatively, bats may be foraging opportunistically and the lower species richness we recorded may reflect greater availability and activity of certain insects at higher temperatures (Clare, Symondson, & Fenton, 2014; Salinas‐Ramos et al., 2015). Further work is required to determine the nutrient content of prey items that are selected by bats under a range of *T*_*a*_ and use bomb calorimetry, and respirometry to determine caloric intakes and expenditures.

The sample size for juvenile bats was low (*n* = 6; 7) and so our conclusions are somewhat speculative. However, demography appeared to play a varied role in MOTU richness and dietary niche breadth depending on the site. During summer in Eglinton, juvenile bats had significantly higher MOTU richness than nonreproductive adults, and juveniles also had the highest dietary niche breadth and, therefore, the most generalist diet. Additionally, juvenile bats in Pureora showed no overlap in dietary niche with lactating females during summer, and adults during winter. Although Arkins (1996) found no difference in *M. tuberculata* diet between age classes on Hauturu, adults and juveniles of several other insectivorous bat species exhibit dietary differences (Adams, 1996, 1997; Hamilton & Barclay, 1998; Rolseth et al., 1994). In some bats, juveniles forage in more open areas due to poor flying skills and likely as a result have different diets to adults (Adams, 1996, 1997; Hamilton & Barclay, 1998; Rolseth et al., 1994). As a result of their poor flying skills, juvenile bats may also capture fewer prey items than adults during the same foraging times (Anthony & Kunz, 1977). Yearling North‐western Crows (*Corvus caurinus*) select a broader range of prey sizes than do adults (Richardson & Verbeek, 1987), and this pattern holds true for some bats (Borkin & Parsons, 2011; Hamilton & Barclay, 1998; Salsamendi et al., 2008). A combination of poor flying and handling skills may lead juvenile *M. tuberculata* to be less “choosy.” These results must be interpreted with caution as our sample size for juveniles was low and we urge future studies to repeat our study with more individuals to confirm our speculative conclusions.

In Pureora during summer, lactating females had lower dietary diversity than other demographic classes. Energy balance and energetic demands of reproduction can affect foraging effort and diet (Anthony & Kunz, 1977; Barclay, 1989; Whitaker, Neefus, & Kunz, 1996). Energetic requirements should be greatest for the demographic with highest energy demands (i.e., reproductive females) (O'Donnell, 2001; Racey & Swift, 1985). The high energetic cost of pregnancy and lactation is more likely to affect foraging strategies compared to males (Kunz, Whitaker, & Wadanoli, 1995; Swift, Racey, & Avery, 1985; Wilkinson & Barclay, 1997). Lactating little brown bats (*Myotis lucifugus*) have narrower diet breadth than other demographics as they are likely to form a “search image” to improve foraging efficiency and increase selectivity (Anthony & Kunz, 1977). Additionally, even when other prey types are available, lactating Mexican free‐tailed bats (*Tadarida brasiliensis*) maintain a narrow diet likely due to water balance requirements (Whitaker et al., 1996). By incorporating more fat‐rich prey items, lactating greater mouse‐tailed bats store an important metabolic water source for when milk production is highest (Levin et al., 2009). In temperate regions, big brown bat feces contained a higher richness of Coleoptera and Trichoptera during late fall and before hibernation, and these are high in linoleic acid, an energy‐rich polyunsaturated fatty acid (Clare, Symondson, & Fenton, 2014; Schalk & Brigham, 1995). Lactating *M. tuberculata* likely face a greater energetic burden than other demographics and may face a greater selection pressure to optimize foraging time by being more “choosy” and selecting high‐quality prey items. Alternatively, a more restricted home range that optimizes foraging effort against energetic gains may be more strongly selected for in females compared to males. The home range requirements of bats are driven by their energetic requirements, which vary according to sex, age, and reproductive status (e.g., Borkin & Parsons, 2011; O'Donnell, 2001; Racey & Swift, 1985). Lactating *M. lucifugus* have 51% smaller home range than males (Henry, Thomas, Vaudry, & Carrier, 2002), and *Pipistrellus pipistrellus* (Racey & Swift, 1985), *Macrophyllum macrophyllum* (Meyer, Weinbeer, & Kalko, 2005), and *Chalinolobus tuberculatus* in the Eglinton Valley (O'Donnell, 2001) all have smaller home ranges than males. This pattern has been attributed to the need for females to visit the roost and feed their young during the night (O'Donnell, 2001; Racey & Swift, 1985).

## 5 CONCLUSIONS

We show that, unlike previous work, *M. tuberculata* incorporate a broad diversity of moths and flies in their diet. Despite the high proportion of moths and flies in their diet, *M. tuberculata* exhibit site‐specific differences in the proportion of prey orders consumed, and dietary diversity, suggesting that certain orders are more influential in certain sites than others. These differences are likely due to site‐specific differences in habitat type and season. We also provide the first evidence of demographic differences in the diet of *M. tuberculata*, with juveniles having the broadest diet, and lactating females the most restricted. Newly available molecular techniques help to unveil new layers of dietary complexity and add finer resolution to understanding behaviors than were possible using previous techniques. Generating an insight into the diverse hunting patterns of generalists may help improve conservation efforts, highlight their crucial role in an ecosystem via stability or biocontrol, and function as proxy for investigating the diversity of an ecosystem itself.

## AUTHOR CONTRIBUTIONS

ZJC, RMB, ELC, and SP designed the project. ZJC performed field work and statistical analysis. JLT, JEL, and ELC extracted DNA and analyzed data. DHB and HFMDO supervised bioinformatics ZJC and JLT wrote the manuscript. SP, RMB, AJHR, and ELC provided editorial assistance.

## DATA ACCESSIBILITY

Molecular data are available via Dryad with full description https://doi.org/10.5061/dryad.825kr16.
